# The morphology of medial malleolus and its clinical relevance

**DOI:** 10.12669/pjms.302.4268

**Published:** 2014

**Authors:** Kun Zhang, Yanxi Chen, Minfei Qiang, Yini Hao, Haobo Li, Hao Dai

**Affiliations:** 1 Kun Zhang, MS, Department of Orthopaedic Trauma, East Hospital, Tongji University School of Medicine, 150 Jimo Rd, 200120 Shanghai, China.; 2 Yanxi Chen, MD, PhD, Department of Orthopaedic Trauma, East Hospital, Tongji University School of Medicine, 150 Jimo Rd, 200120 Shanghai, China.; 3 Minfei Qiang, MS, Department of Orthopaedic Trauma, East Hospital, Tongji University School of Medicine, 150 Jimo Rd, 200120 Shanghai, China.; 4 Yini Hao, MS, Department of Orthopaedic Trauma, East Hospital, Tongji University School of Medicine, 150 Jimo Rd, 200120 Shanghai, China.; 5 Haobo Li, MS, Department of Orthopaedic Trauma, East Hospital, Tongji University School of Medicine, 150 Jimo Rd, 200120 Shanghai, China.; 6 Hao Dai, MS, Department of Orthopaedic Trauma, East Hospital, Tongji University School of Medicine, 150 Jimo Rd, 200120 Shanghai, China.

**Keywords:** Medial malleolus, Tibia, Tomography, X-ray computed, Imaging, three-dimensional

## Abstract

***Objective:*** To provide morphological data of medial malleolus to decrease the possibility of posterior tibial tendon injury and inadvertent ankle penetration.

***Methods:*** Computed tomography scans of the ankle in 215 patients were reviewed. Then parameters in the 3-D reconstruction images were measured by three independent, qualified observers on two separate occasions.

***Results:*** The average angle between tibia plafond and the articular facet of the medial malleolus was 55.88±4.11°. The distance from the most anterior point of the anterior colliculus to the center of the intercollicular groove was 11.68±1.13 mm. And the average angle between the bimalleolar axis and the articular facet of the medial malleolus was 76.61±2.04°. Significant differences were observed in the distance from the most anterior point of the anterior colliculus to the center of the intercollicular groove between males and females. (*P*<0.05) All of the parameters exhibited moderate to excellent intra-class correlation coefficient (ICC).

***Conclusions:*** According to this study, the insertion angle is much smaller than previously believed, and adequate space only exists for two 4.0-mm screws in some large cases. The second screw will probably be near the posterior tibial tendon, especially in some small cases.

## INTRODUCTION

Ankle fractures are among the most common surgically treated fractures.^1^ As the fixation of medial malleolus is critical for ankle stability in bimalleolar fractures,^2^ open reduction and internal fixation is generally recommended.^3^^,^^4^ In addition isolated medial malleolar fractures with joint incongruence may also be treated surgically.^2^^,^^5^

As various techniques were applied in the treatment of medial malleolus, complications such as hardware-pain emerged which can be due to prominent implants, impingement of deeper soft-tissue and inadvertent ankle penetration.^5^^,^^6^^,^^7^ Although outcomes after fixation were usually good,^1^^,^^8^^,^^9^ complications related to painful implants were relatively high and may result in reoperation for implant removal.^6^^,^^7^ Therefore, a good knowledge of the morphology of medial malleolus is of crucial importance to minimize prominence of medial screws and improve patient outcomes. However, little data is available concerning the morphology of the medial malleolus.

The objective of the study was to provide morphological data of medial malleolus to decrease the possibility of posterior tibial tendon injury and inadvertent ankle penetration.

## METHODS

***Subjects: ***Institutional ethical approval for this study was obtained from the Ethics Committee of the hospital, and conforms to the provisions of the Declaration of Helsinki. (East Hospital Ethics Committee, Ethics number 2012-020). The patients were collected from the foot and ankle clinic of our hospital and informed consent was obtained. Patients were excluded if they had a history of ankle fracture or pilon fracture confirmed by radiological examination or surgery. Patients with congenital or acquired malformation, rheumatoid arthritis, osteoarthritis or a history of bone tumor of ankle were also excluded. Patients less than 20 years old or older than 65 years were excluded to avoid skeletal immaturity or degeneration. Finally, 215 patients were enrolled in this study, with 113 men (with 113 ankle joints) and 102 women (with 102 ankle joints). There were 96 left and 119 right ankle joints. The average age was 40.5 years (range, 20 to 65 years).

***Image acquisition and post processing: ***The thin-slice CT images (DICOM 3.0 format) of all the patients scanned by 16-row spiral CT (Light Speed, GE, USA) were collected. Main CT scanning parameters were as follows: thickness, 0.625 mm; voltage, 120 kV; current, 200 mA; image matrix, 512^.^512.

**Fig.1:**
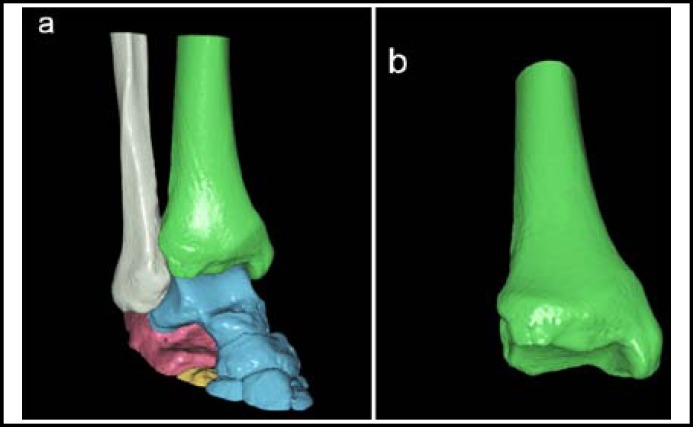
Three dimensional image post processing. (a) Three dimensional SSD images were generated, and all component bones were distinguished from tibia. (b) Then all bones except tibia were hidden

The thin-slice CT axial images of all research subjects were firstly uploaded to picture archiving and communication system (PACS), then these data were imported into the digital orthopaedic clinical research platform (SuperImage orthopaedics edition 1.0, Cybermed Ltd, Shanghai, China) via removable storage devices. On this platform, three-dimensional (3-D) images were generated by performing surface shaded display (SSD) with a bone algorithm at 0.625 mm slice thickness. All component bones of ankle joints were distinguished by computer in 3-D images (Fig.1). Then parameters in the 3-D reconstruction images were measured and calculated.

***Measurements and calculations: ***Based on the measurement module which was inside the digital research platform, we took the following steps. Firstly, on the distal tibia plafond, the turning point of posterior malleolus and medial malleolus (point A), the turning point of medial malleolus and anterior ankle (point B) and the top point of the lateral margin (point C) were selected to define the cross-section (plane ABC) which corresponded to the axial plane at the level of the plafond.

Secondly, connected point A and B, and draw a plane perpendicular to line AB through the tip of anterior colliculus of medial malleolus (point D). On this plane, the angle between tibia plafond and the articular facet of the medial malleolus (∠α) was measured. Next, plane E was determined through the apical point of the intercollicular groove of the medial malleolus which is parallel to the tibia plafond. 

**Fig.2:**
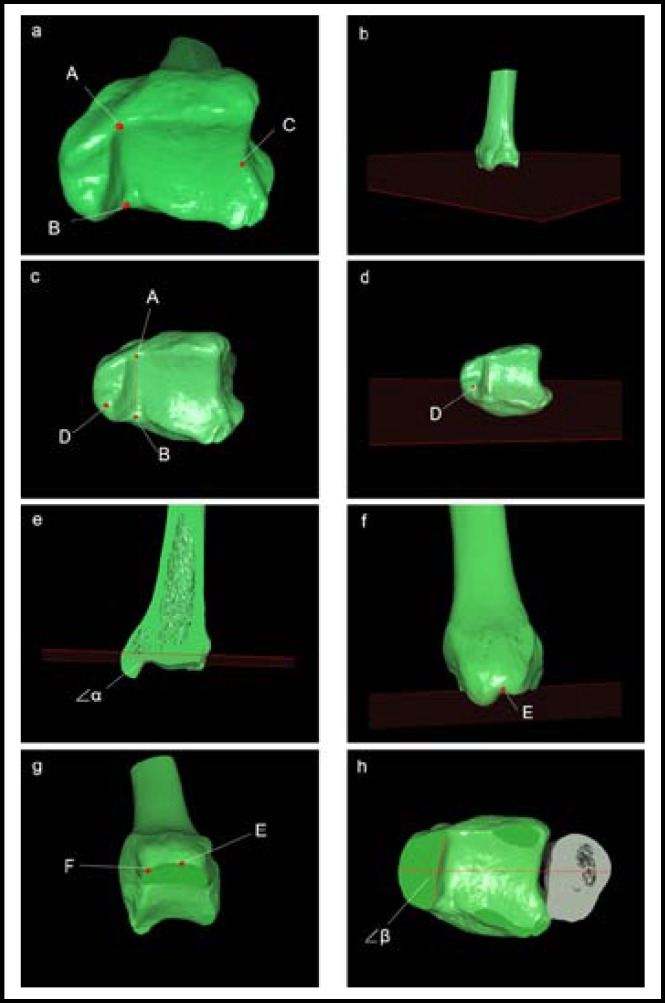
Measurement on 3-D images. (a, b) The cross-section which was corresponded to the axial plane at the level of the plafond was defined by selecting three points: the turning point of posterior malleolus and medial malleolus (point A), the turning point of medial malleolus and anterior ankle (point B) and the top point of the lateral margin (point C). (c, d) Connect point A and B, and draw a plane perpendicular to line AB through the tip of anterior colliculus of medial malleolus (point D). (e) On plane D, the angle between tibia plafond and the articular facet of the medial malleolus (∠α) was measured. (f) Plane E was determined through the apical point of the intercollicular groove of the medial malleolus which is parallel to the tibia plafond. (g) On plane E, the distance from the most anterior point of the anterior colliculus to the center of the intercollicular groove (line EF) was obtained. (h) Then fibula was redisplayed and the angle between the bimalleolar axis and the articular facet of the medial malleolus (∠β) was measured.

Finally, on this profile, the distance from the most anterior point of the anterior colliculus to the center of the intercollicular groove (line EF) was obtained. Then fibula was redisplayed and the angle between the bimalleolar axis and the articular facet of the medial malleolus (∠β) was also measured. (Fig.2).

The measurements were performed on two separate occasions by three independent, qualified observers. All observers were blinded to the other’s analysis. The average was taken as the final data.

***Statistical analysis: ***SPSS 18.0 (SPSS Inc, Chicago, IL, USA) was used for statistical analysis. The parameters between males and females, and between left and right limbs were compared using the two-samples t test. The intra-class correlation coefficient (ICC) was used to assess intraobserver and interobserver reliability. *P*＜0.05 was considered to be statistically significant.

## RESULTS

The average angle between tibia plafond and the articular facet of the medial malleolus (∠α) was 55.88±4.11° (range 47.43-66.30°, 95% confidence interval [CI]: 55.10-56.66). The distance from the most anterior point of the anterior colliculus to the center of the intercollicular groove (line EF) was 11.68±1.13 mm (range 9.19-14.73 mm, 95% confidence interval [CI]: 11.47-11.90). And the average angle between the bimalleolar axis and the articular facet of the medial malleolus (∠β) was 76.61±2.04° (range 71.83-81.17°, 95% confidence interval [CI]: 76.32-76.90).

**Fig.3:**
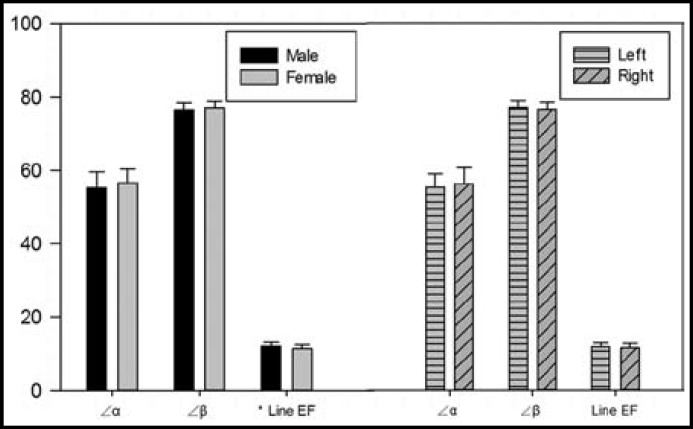
*Significant differences were observed in the distance from the most anterior point of the anterior colliculus to the center of the intercollicular groove (Line EF) between males and females. (*P*<0.05)

Significant differences were observed in the distance from the most anterior point of the anterior colliculus to the center of the intercollicular groove (Line EF) between males and females. (*P*<0.05, Fig.3).

The reliability of all parameters and the intra-observer reliability between three observers is shown in Table-I.

**Table-I:** Intra-observer and inter-observer reliability of all parameters

	*Observer 1*	*Observer 2*	*Observer 3*	*ICC*_1_	*ICC*_2_	*ICC*_3_	*ICC*_12_	*ICC*_13_	*ICC*_23_
α (°)	55.94±4.17	55.87±4.11	55.82±4.08	0.99	0.99	0.99	0.98	0.99	0.99
β(°)	76.71±1.93	76.50±2.37	76.50±2.02	0.79	0.68	0.77	0.89	0.91	0.88
Line EF	11.66±1.16	11.71±1.15	11.68±1.13	0.90	0.93	0.90	0.96	0.98	0.96

α, the angle between tibia plafond and the articular facet of the medial malleolus; ∠β, the angle between the bimalleolar axis and the articular facet of the medial malleolus; Line EF, the distance from the most anterior point of the anterior colliculus to the center of the intercollicular groove.

ICC_1_, ICC_2_, ICC_3_, intraclass correlation coefficient for the first, second and third observer; ICC_12_, ICC_13_, ICC_23_, interclass correlation coefficient between the first and second, between the first and third and between the second and third observer, respectively.

Reliability is excellent if ICC is greater than or equal to 0.75, moderate if between 0.4and 0.74, poor if less than or equal to 0.4.

## DISSCUSION

All the parameters exhibited moderate to excellent ICC. It demonstrates that the morphological measurement on 3-D CT post processing images can provide a reproducible and stable data set. Besides, it’s easier to get a large amount of CT data than to collect a large sample of cadaver specimens. In this study, surface shaded display was applied. The 3-D rendering technique was the first applied to medical imaging and was mainly applied in orthopaedics because of its superiority for bony surface reconstructions.^10^^-^^12^ It can generate distinct surfaces so as to facilitate clinical measurements.^13^ Limitations are inevitable when measuring on X-ray or CT films. Errors may appear because of wrong projective angle of the tube, ankle swelling and image overlap on plain films. On CT films, parameters can only be measured on single slice. While using 3-D CT post-processing techniques, images can be rotated freely, and parameters can be measured in the 3-D scene.

The intersection angle we measured between the tibia plafond and the articular facet of the medial malleolus can be used to facilitate a safer and more convenient operation of medial malleolus fractures. Scholars had indicated a small medial clear space and superior tibiotalar clear space,^14^^,^^15^ thus there is little room for error fixing medial malleolus. Wrong insertion angle may result in hardware-pain which can be due to prominent implants, impingement of deeper soft-tissue and inadvertent ankle penetration.^5^^,^^6^^,^^7^ Previous study determined it 65.5° measured by X-ray, but the limitation of measuring 3D structure via 2D method was also exposed by the author.^16^ Although the average angle between the bimalleolar axis and the articular facet of the medial malleolus was 76.61°, which corroborates the 15° internal rotation of mortise view in X-ray films. A big difference between two angles was highlighted by this study. The 3D measurement results of this research suggested the angle was 55.88°. Knowledge of the angle should reduce the risk of the penetration through articular surface. The results can also provide a quantitative standard to evaluate the reduction during and after operation. What’s more, no significant differences exist between males and females and between left and right limbs. Therefore, the implanted angle could be determined by measuring the opposite side and regardless gender.

Femino et al.^5^ indicated that medial malleolar screws placed in the posterior colliculus resulted in tendon abutment in all specimens and in tendon injury in half. Although various techniques were applied in treatment of medial malleolus, two 4.0-mm partially threaded cancellous lag screws remain a standard method to treat medial malleolar fractures.^2^^,^^6^ Questions were raised that is there enough space to insert two or more screws in the anterior colliculus and intercollicular groove? The distance from the most anterior point of the anterior colliculus to the center of the intercollicular groove was 11.68 mm (9.19-14.73 mm). According to this study, adequate space only exists for two 4.0-mm screws in some large cases, and not for more. The space between two screws should be as small as possible. Meanwhile, it is worth noting that, if a triple guide is used which has a 5 mm interval, the middle screw will probably abut the posterior tibial tendon, especially in some small cases. As such one has to be careful while operating. 

There are some limitations of the study. The cross-sections in this study were redefined, which were presented for the first time. Therefore, the veracity and rationality need continuous further study and improvement. However, the study is the first to provide detailed data of medial malleolus based on 3-D measurement. Knowledge of the results may help to decrease the possibility of posterior tibial tendon injury and inadvertent ankle penetration.
